# Functions and Activities Missed by Nurse Managers, Their Antecedents and Consequences: Findings From a Qualitative Study

**DOI:** 10.1155/jonm/4992301

**Published:** 2026-06-29

**Authors:** Stefania Chiappinotto, Gulcan Taskiran Eskici, Francesca Miorin, Alvisa Palese

**Affiliations:** ^1^ Department of Medicine, University of Udine, Udine, Italy, uniud.it; ^2^ Department of Nursing Management, Ondokuz Mayis University, Samsun, Turkey, omu.edu.tr; ^3^ Distretti Sociosanitari Carnia e Gemonese,Val Canale e Canal del Ferro, Azienda Sanitaria Universitaria Friuli Centrale, Udine, Italy

**Keywords:** antecedents, consequences, missed nursing management activities, missed nursing management functions, nurse managers, nursing management, qualitative study

## Abstract

**Objective:**

To understand the phenomenon of Missed Nursing Management functions and activities in the daily practice of nurse managers (NMs) and to identify its antecedents and consequences.

**Design:**

A descriptive qualitative study conducted according to the COnsolidated criteria for REporting Qualitative research guidelines.

**Methods:**

A purposive sample of 22 Italian nurses from different roles—NMs, their clinical nurses, nurse executives, representatives of NM associations, and nurse educators—were identified and individually interviewed face‐to‐face in 2023. The data were thematically categorized using both inductive and deductive approaches.

**Findings:**

Four Missed Nursing Management functions have emerged: (1) “Missing systemic planning and monitoring”, (2) “Missing effective presence in the clinical environment”, (3) “Missing coordination and continuous alignment with expected goals” and (4) “Missing to promote the development of the department, staff and the profession”. Several antecedents were identified at the (1) macrolevel: “Systemic challenges such as the shortage of nurses and postpandemic recovery”, “Strategic systemic uncertainties”, “Inadequate role recognition”; (2) exolevel: “Lacks in tailored training and professional development opportunities”, “Lack of independence in decision making”; (3) mesolevel: “Lack of structural and organizational support”, “Lack of coordination”; (4) microlevel: “Ambiguities in role expectations”, “Bureaucratic burden”, “Lack of team cohesion and a feeling of loneliness”, “Disjointed information” and (5) NM level: “Chronic time scarcity”, “Lack of experience”, “Attitudes”, “Sense of disengagement”. Missed Nursing Management consequences were reported at NM, staff, unit and patient/family levels.

**Conclusions:**

Essential nursing management functions, such as planning, supervision, visibility, coordination and professional development, are missed or postponed by NMs, as activities like resource management remain prioritised for daily unit functioning. Antecedents influence the phenomenon across multiple levels, indicating the need for system‐wide interventions, while consequences primarily affect the microsystem, including NMs, teams, organisational performance and potentially care quality.

**Implication for Nursing Management:**

These results inform management by highlighting the need to protect NMs’ time for core managerial functions, strengthen their visible presence in clinical settings, and reduce bureaucratic burden. From an organisational and policy perspective, the study supports investment in role clarification and decision‐making autonomy, as well as workforce policies that address structural staffing shortages and strengthen coordination across system levels. Moreover, it is essential to develop strategies that enhance leadership competencies and support NMs to fully perform their leadership role, creating an environment that enables them to fulfil their strategic and support responsibilities.

## 1. Introduction

Missed Nursing Care (MNC) is a widely recognized indicator of nursing quality and patient safety [1], defined as any aspect of required care that is omitted or delayed [2, 3]. Evidence on the MNC phenomenon has increased in recent years, documenting its occurrence, the activities most frequently missed at the bedside [4, 5], and the consequences for patients, nurses and healthcare services [6, 7]. More empirical data have also been provided regarding the reasons underlying MNC, identifying them at the nurse, nurse manager (NM), unit, and system levels [8, 9]. However, as recently highlighted, most evidence has originated from the perspective of clinical nurses and only in a few cases from that of NMs, who may act as supervisors of overall care quality [10], as promoters of strategies to minimise MNC [11], and as possible sources of missed care when staffing decisions, workload distribution, prioritisation processes and support for clinical staff are inadequate [12, 13]. Moreover, NMs, like nurses, may delay or omit important functions or activities expected within their role. All professions are exposed to this risk, as also documented in recent literature on physicians [14]. However, to date, no studies have specifically examined which functions or activities NMs sacrifice in their daily practice by omitting or postponing them, nor the reasons and consequences of such phenomena. By addressing this gap through the exploration of functions or activities missed or postponed by NMs, this study aims to shift the scientific debate from missed care activities to missed nursing managerial functions or activities, and to generate knowledge that may inform tool development and strategies aimed at measuring, preventing and minimising its occurrence.

### 1.1. Background

NMs have been defined as “*directly responsible for the day-to-day operations of one or more organisational units. His or her major responsibility is implementing policy, handling problems and disseminating information about patient problems and the allocation of human resources*” [15]. At the unit level, NMs play a pivotal role in leading and negotiating resources, allocating them across shifts and supervising organisational processes that influence staff satisfaction and performance [16]. They are the closest managerial position to both nurses and patients and are responsible for several functions that may be operationalised in different, interrelated activities [16, 17]. Conceptually, functions include a range of responsibilities reflecting the purpose of the role—such as planning, organising, directing and controlling—whereas activities refer to the operational actions that actualise these functions [18]. For instance, preparing staff schedules is the concrete activity within the broader function of resource planning.

To date, there is no shared framework that defines the distinction between the functions and activities of NMs. However, Witges and Scanlan [19], drawing on Full‐Range Leadership Theory, proposed a clear conceptual separation between transactional operational activities (such as human resources management and performance evaluation), which are necessary for the daily functioning of the unit, and transformational leadership functions (such as building trust, promoting empowerment, innovation and critical thinking), which are oriented towards the development of staff, organisational culture and change. In summary, the former may be considered operational activities, while the latter may be considered functions that define the scope of the NM’s role.

Overall, according to the available literature, NMs are expected to ensure effective resource planning and management, thus contributing to the achievement of optimal results for both the organisation and the patients [20]. Moreover, they are expected to periodically assess nurses’ performance, educational needs and the quality of care to create a culture of safety [21–23]. On a daily basis, NMs are responsible for planning staffing and skill mix for optimum care while maintaining focus on the strategic goals of the organisation and preventing MNC [6, 24, 25].

Furthermore, NMs are key to creating an environment for teamwork among nurses in the unit and encouraging collaboration [5, 26, 27]. In addition, NMs are expected to create a culture of open communication, transparency and speaking up, providing an opportunity for nurses to report MNCs in a supportive and nonpunitive environment [28]. Their key function is also to enhance and sustain nurses’ work motivation to prevent burnout and job dissatisfaction, even in resource‐limited settings [29]. In this context, their orientation should focus on people and relationships rather than solely on completing tasks and financial goals [30].

All the abovementioned functions and activities, although conceptualised differently, have been consistently associated, when fully and timely performed, with reducing or preventing MNC [26, 31–34]. However, all of these functions and activities may be missed or postponed in daily practice due to the high workloads of NMs, their span of control (e.g., when they lead large teams) or other reasons. Therefore, although MNC is traditionally defined as omissions or delays in direct patient care activities [2, 35], there is limited understanding of which managerial activities or functions NMs miss, why these omissions occur, and what consequences they entail. While attempts have been made to translate the MNC concept into the educational domain, examining missed functions among nurse educators, along with their underlying reasons and consequences [36], the managerial perspective remains underexplored.

## 2. The Study

### 2.1. Research Questions and Aim

The following research questions were addressed: (1) What functions and activities are missed or postponed by NMs in daily practice? (2) What are the underlying reasons? (3) What are the consequences? Therefore, the main aim of this study was to understand the phenomenon of Missed Nursing Management functions and activities in the daily practice of NMs and to identify their antecedents and consequences.

### 2.2. Design

A descriptive qualitative study design [37] based on individual interviews was performed in 2023. This study design was selected because it is suitable for exploring emerging concepts related to nursing phenomena [38] and according to two main underlying principles:

(1) Theory diffusion, in which conceptual work established in one field (specifically, MNC as documented in clinical nursing practice) is transferred to another field (that concerning the managerial aspect of the NMs’ role) [39].
(2) An open and exploratory perspective, without restricting the analysis to a specific level (NMs’ functions or activities), allowing findings to emerge inductively from the data according to the interrelations between functions and activities [16, 17] and the lack of conceptualisation available in the field of the NM role [20, 40, 41].

### 2.3. Sampling and Setting

A purposive sampling method [43], aimed at maximising the variability of participant experience with respect to the phenomenon under study [44], was adopted. As the study was designed according to the principles of diffusion of theory and/or research [39], the sampling method first used by pioneering researchers in the field of MNC [2] was considered, mirroring the same composition to best reflect the complexity of nursing practice [2]. Five target groups of participants were identified, selected from different professional levels: (1) NMs, offering direct perceptions of the phenomenon; (2) clinical nurses, contributing the viewpoint of those who directly experience missed or postponed NM activities; (3) nurse executives, responsible for supervising NMs and perceiving issues in their role implementation; (4) nurse educators, responsible for educating NMs in postgraduate and continuing education courses and (5) representatives of NM associationS, providing perspectives regarding the expected role and issues in fully implementing it. This approach, encompassing different hierarchical levels (clinical nurses, NMs and nurse executives) and a cross‐cutting perspective (involving nurse educators and NM associations), was chosen to strengthen analytical depth, enable triangulation of data sources and enhance the credibility of the findings [45]. Comparing and integrating perspectives across these groups helped to explore how managerial missed functions or activities are perceived.

A large healthcare trust with several hospitals (one academic) located in a region of North‐East Italy was approached. Eligible participants included: (a) NMs at the unit level and their clinical nurses; (b) nurse executives supervising NMs in the different hospitals involved; (c) nurse educators across multiple universities in the region and (d) representatives of NM associations also operating in the region. Participants were included if they had worked for at least 6 months in their current role and agreed to participate in the study.

One researcher (AP) directly contacted the participants nominated by the research team via e‐mail and invited them to participate in the study. Recruitment continued until data saturation [46] was reached (22 participants) as independently judged by three researchers (MF, SC and AP). All participants were invited to participate by providing written informed consent after receiving full information regarding the study aims and the data collection process. All participants also gave permission to be audio‐recorded.

### 2.4. Data Collection

To obtain participants’ in‐depth perceptions and experiences [38], an open‐ended face‐to‐face interview guide was developed. A short data collection form covering demographic and professional information (e.g., age, gender and education) was designed, followed by an interview guide (Table 1) to explore managerial functions or activities that were missed or postponed. Specifically, given the different possible levels of the managerial function, as expressed by the different roles and levels within organisational systems (e.g., [15–19]), the unit level was targeted. Therefore, the questions (see Table 1) focused on Missed Nursing Managerial activities or functions at the unit level.

**TABLE 1:** Interview guide: questions.

Questions
Demographic and professional data
Warming up
Concerning the concept of Missed Nursing Care, that have been extensively documented in the nursing practice, the interviewer shares the definition to create a common understanding.
Prompting
Missing or delaying some expected functions or activities may happen also to NMs at the unit level… Do you have any experience with this?
Core questions
What activities are missed or postponed in/by the NMs at the unit level according to your experience? Can you report some examples?
What are the reasons for these missed or postponed managerial functions or activities according to your experience? Can you report some examples?
What are the main consequences that these missed or postponed managerial functions or activities have according to your experience? Can you report some examples?
Do you have additional comments/insights in this field?
Ending
Please express your degree of satisfaction in the current managerial role.^1^
From 0—none to 10—maximum degree of satisfaction.

*Note:* NMs: nurse managers.

^1^Nurses were not asked to report their satisfaction in the managerial role given their clinical role.

The guide was first piloted (MF) with a master’s student and an NM to assess its feasibility and clarity; no changes were requested, and the data collected were not included in the final analysis. All potential participants contacted by the senior researcher (AP) agreed to take part in the study; interviews were scheduled at times and locations convenient for the participants. For each interview, two researchers (SC and MF) were present: one (MF) provided expertise related to the NM role, and the other (SC) oversaw data collection procedures and took field notes. Each interview lasted approximately 1 h.

### 2.5. Data Analysis and Rigour

The audio‐recorded interviews were transcribed verbatim (MF), anonymised, and consecutively enumerated. The process was carried out immediately to gain insights and assess saturation [46]. Researchers then discussed their preconceptions regarding the phenomenon under study [43] and involved an additional researcher (GTE), based on her experience in the field of MNC, to enrich the data analysis, double‐check the process and contribute during the reporting phase.

First, the transcripts were carefully and independently read by three researchers (MF, SC and AP) to obtain a global view of the perspectives. They subsequently read and reread the transcripts, analysing the text sentence by sentence to ensure comprehensive understanding. The transcripts were translated to enable full participation of the internationally based research team (see authors) [47]. The approach was both inductive and deductive. Initially, two researchers (MF and SC) conducted an inductive thematic analysis based on the main research questions [48]: (a) missed nursing management functions and activities of NMs, (b) antecedents (i.e., underlying factors or reasons) and (c) consequences. The researchers (MF and SC) independently performed initial coding, separating the main segments: those related to Missed Nursing Management functions or activities, antecedents and consequences.

Themes as functions and subthemes as activities were identified from the data, and any disagreements were resolved through discussion among all researchers, including during an in‐person meeting held in May 2025 (Italy). Explanatory quotes were identified and extracted to support the findings with illustrative examples. Similarly, antecedents and consequences were identified. During the analysis, researchers realised that the distinction between missed functions and activities and their antecedents was not always clear, as some quotes could reasonably be situated at both levels depending on how they are enacted in practice. For example, lack of coordination was described as both a missed or postponed activity and as an antecedent, reflecting structural or organizational conditions that constrain effective management. In fact, when NMs consistently fail to share emerging problems, critical information, or decision‐making processes with other managers, this lack of coordination can also be seen as a missed function or activity. In such cases, the issue is no longer a contextual condition but becomes a specific omission within the managerial role, with potential consequences. Thus, the segments and their categorisation into missed functions or activities, antecedents or consequences closely followed the participants’ narratives.

Once this first phase was concluded, a deductive approach was used. Specifically, antecedents and consequences were categorised according to the levels identified by Jones et al. [49]: macrosystem (e.g., social culture, subcultures and ideologies), exosystem (e.g., education, legislative and regulatory bodies), mesosystem (e.g., clinical departments, administration, finance and human resources), microsystem (i.e., the direct care setting) and the level of NMs themselves.

Overall, the rigour of the study was ensured by enacting the strategies summarised in Supporting Table 2, while the audit trails are available in Supporting Tables 3 and 4. Specifically, to ensure maximum credibility in the categorisation process, the narratives were read several times, and the categorisation was agreed upon during a face‐to‐face meeting. In addition, the findings were presented in two meetings with more than 50 local NMs and discussed to enhance the validity of the interpretations.

### 2.6. Ethical Issues

Approval was obtained from the Internal Review Board of the University of Udine, Italy (RIF Prot IRB: 049/2022; Tit III cl 13 fasc.8/2022). Each participant was informed of the aims of the study and provided written informed consent. Participation was voluntary, with no incentives offered, and participants could withdraw from the study at any time without consequence. Confidentiality was strictly maintained throughout the study. Participants were assured that their shared experiences would remain confidential and were encouraged to respond as openly and honestly as possible. To ensure anonymity, all personal identifiers were removed from the verbatim transcripts, and each participant was assigned an identifying code. Quotations were indexed using codes (e.g., *P* for participant) with a numerical identifier. Confidentiality was preserved at all stages of the research.

## 3. Results

### 3.1. Participants

A total of 22 nurses participated in the study: 5 NMs, 5 nurse executives, 5 nurse educators, four representatives from NM associations and 3 clinical nurses (Table 2). Participants ranged in age from 24 to 66 years and were predominantly female (13 of 22). Their nursing experience ranged from 2 to 42 years, while managerial experience ranged from 1 to 29 years. Among participants in managerial roles, self‐reported job satisfaction ranged from 5 to 10, with a mean and median of 8 out of 10.

**TABLE 2:** Main characteristics of participants.

ID	Gender	Age (years)	Current role	Experience as a nurse (years)	Experience in managerial role (years)	Satisfaction in the managerial role (from 0 none to 10 maximum)
1	M	58	Nurse Executive	40	29	10
2	M	66	Nurse Manager	42	17	10
3	M	47	NM Association	22	9	6
4	F	55	NM Association	35	23	10
5	F	57	Nurse Manager	37	17	5
6	F	58	Nurse Executive	37	30	8
7	F	41	Nurse Educator	19	4	7
8	M	37	Nurse Educator	15	5	10
9	M	35	Nurse Educator	9	1	8
10	M	33	Nurse Educator	10	4	7
11	F	42	Nurse Educator	19	4	6
12	F	50	Nurse Manager	31	18	9
13	F	37	Nurse Executive	15	4	8
14	M	34	NM Association	12	3	8
15	F	47	NM Association	24	7	7
16	F	26	Clinical Nurse	3	NA	NA
17	F	49	Nurse Manager	27	14	7
18	F	24	Clinical Nurse	2	NA	NA
19	M	59	Nurse Executive	33	26	8
20	M	48	Nurse Manager	23	13	9
21	F	59	Nurse Executive	40	14	10
22	F	30	Clinical Nurse	8	NA	NA

*Note:* NA: nurses were not asked to report their experience and satisfaction in the managerial role given their clinical role.

Legend: ID: identifier code; F: female; M: Male; NM: nurse manager.

### 3.2. Missed Nursing Management Function and Activities

Four main missed functions (themes) emerged, along with 13 activities (subthemes), as shown in Table 3 and Supporting Table 3. The first concerns the perception of “*Missing systematic planning and monitoring*”; that is, failing to plan unit activities, determine its direction and monitor results effectively. Specifically, interviewed nurses reported that NMs fail to identify goals and priorities (“*I can’t plan*”, P21) and do not use data and indicators that enable performance monitoring (“*There is a big gap in data management*”, P15).

**TABLE 3:** Missed nursing management: functions, activities and quotes.

Themes: Functions	Subthemes: Activities	Quotes: Examples
Missing systemic planning and monitoring	Missing setting unit goals and priorities to ensure that the strategic mission is addressed	“Having a management that tells you ‘This is the project, and we are working for it,’ communicates that and clears away the doubts… would be more accepted by the employees than working without knowing anything.” (P15)
	Missing using data and indicators	“There is a big gap on data management.” (P15)

Missing effective presence in the clinical environment	Missing being present, visible and ensuring proximity	“Definitely the nursing management… lacks in being present…” (P17)
	Missing taking care of my team	“So also, to take care of people of work groups.” (P6)
	Missing taking care of individual emotional and relational needs	“A worker who is serene, is motivated… works more effectively and efficiently, and this is often, in my opinion, forgotten.” (P22)
	Missing seeing patients	“Instead, you tend to neglect them more and more… I am not able to see the patients for days… this is something that makes me sick.” (P4)

Missing coordination and continuous alignment to the expected goals	Missing managing information flows and deadlines	“So, if you can curb that little daily drama every time you have something new within the department, however, you have to share it first, you have to have the professionals on your side, in my opinion. That is one of the things that is omitted, right now eh, where I have been working, these last 2, 3 years.” (P18)
	Missing communicating and sharing information	“That’s why we need such moments to meet, which are not wasted time. If, on the other hand, the meeting is made to say: from tomorrow you have to do this and that, because I was told so… this is a mechanism that nobody would like. However, this is what we experience in our everyday life.” (P2)
	Missing supervising and controlling	“We give rules and then everyone makes up their own rules within the departments.” (P19)
	Missing holding meetings with middle managers	“The thing I miss the most is the contact with the intermediate structures, let’s call them that, which are the coordinators.” (P1)

Missing to promote unit, staff and professional development	Missing dedicating time to students	“I think it is difficult to find the space to cultivate more of a relationship with students.” (P9)
	Missing planning effective educational strategies and acting as a coach/mentor	“Another aspect that, let’s say, I find that the coordinator miss is what I was telling you earlier, to follow up on the staff introduction stages, because you can it to a mentor in whom you have utmost confidence, that’s perfectly fine, however it’s not like the newly hired nurse has to be constantly following a nurse all the time because that’s his nurse, however it’s certainly important for the coordinator to be proactive, to get informed, to go there, try to see, work, maybe with a trivial excuse, together with the newly hired person saying “come I’ll explain this thing, let me see how you do this thing,” in such a way that you understand how much this person is in line with the directions because if I ask “tell me how this patient is today and what have we done or what does he need” or go and listen to the handovers…” (P17)
	Missing self‐reflection and learning	“Meaning that one’s training activity at this time is a bit missed, in the sense that you devote time to the organization, you devote time to your colleagues, you are focused on that and you have a bit of a hard time engaging in activities for yourself, for your professional growth, to enrich your skills. And so if all the legislation comes and you have to study it, everything that is related to your potential development instead becomes less important, because you are focused on the others than on your role anyway.” (P13)

*Note:* *P*: participant.

The second, “*Missing effective presence in the clinical environment*”, reflects the widespread perception that NMs are neither physically nor relationally present in the clinical setting. This lack of presence undermines the effectiveness of their role, which is believed to be strongly linked to proximity to staff and patients. Nurses reported that NMs have little or no time to be physically present or visible on the unit, as they are constantly pulled away by numerous nonclinical responsibilities (“*She [the NM] never comes to the ward*”, P18). Participants also noted that NMs lack the opportunity to care for the team as a whole—its well‐being, functioning, integration, internal communication and professional needs (“…*to respond to the needs of the professionals*…”, P22). In addition, they reported that NMs lack the opportunity to attend to individual nurses, particularly regarding their emotional and relational needs or vulnerabilities (“*I regret that I was not able to give a personal response*”, P13), for example, when trying to organise shift schedules appropriately. Some participants also noted that NMs completely missed contact with patients: “*I am not able to see the patients for days*…” (P4).

The third missed function, “*Missing coordination and continuous alignment with expected goals*”, refers to NMs’ daily tasks in coordinating clinical processes, fostering relationships and team dynamics and continuously supporting alignment with organisational expectations. Participants reported that NMs find it difficult to manage information, meet deadlines and deal effectively with new data (“*Clinical nurses do not receive some information in a timely manner*”, P16). They are also often unable to share information to deepen understanding, stimulate discussion, or provide explanations (“…*a time for confrontation with staff*… *a chance to explain*…”, P17). They also have difficulty involving staff in decision‐making before implementing a new intervention, both informally and through formal structured processes. Many activities need to be delegated (“…*the emergency trolley*…”, P16), but NMs often fail to follow up, control, or monitor these delegated tasks. In addition to these internal challenges, there is also a lack of horizontal coordination among NMs across units. This coordination rarely takes place, either informally or through structured processes (“…*meetings are rarer*…”, P6).

The fourth missed function, “*Missing to promote the development of the department, staff and profession*”, highlights that NMs feel unable to give adequate attention to continuous improvement—not only of their units, but also of the nursing staff, the profession itself and the students present in the department. Participants reported that NMs must give up time they could devote to students (“…*It’s secondary*…”, P10). They find it difficult to build relationships, provide support, or make a meaningful contribution, for example, by participating in student mentoring. Furthermore, NMs are often unable to plan educational strategies tailored to staff needs or engage in mentoring activities, even for those on postgraduate programmes (“I *do not think educational needs are adequately supported*”, P22). This lack of focus on development also affects NMs themselves. Participants reported that NMs have little time for personal learning, professional development, or reflective practice (“*You dedicate your time to the organisation*… *you find it hard to do activities for yourself*…”, P13).

### 3.3. Antecedents

Overall, 15 antecedents were identified across five levels (Table 4; Supporting Table 4). At the macro level, “*Systemic challenges such as the shortage of nurses and postpandemic recovery*” have created a difficult environment in which NMs are busy managing human resources and ensuring standards of daily care. As a result, they often sacrifice time for functions beyond staffing and resource allocation. In addition, the ongoing efforts required due to the pandemic (“*Maybe COVID has created difficulties*”, P3) and the lack of a full recovery have led them to neglect management functions that normally fall within their scope of practice. At the same time, “*Strategic systemic uncertainties*” were also cited as a contributing factor to MNM. Hospitals often do not have a clearly defined strategic direction, or if there is one, it is often changed by political or external influences. This forces NMs to work mainly on a day‐to‐day basis and focus on immediate demands *(“*…*you live in the contingency of the day-to-day*”, P19). Finally, “*Inadequate role recognition*” was mentioned, as the limited powers given to NMs undermine their ability to make a difference and make every effort more arduous (“…*role recognition that has gone missing*”, P6). This constant effort requires a lot of energy, which is then diverted from other important functions that are left unaddressed.

**TABLE 4:** Missed nursing management: levels, antecedents and main quotes.

Level	Antecedents	Main quotes
Macrolevel	Systemic challenges (shortages and postpandemic recovery)	“Maybe COVID has created difficulties, maybe so much has been asked, maybe people are tired of earning little, so many causes.” (P3) “A situation whereby they live a little bit like the nurses who work in the clinic, in my opinion, with maybe not an adequate number of workers. An inadequate number compared to the size and complexity of the facilities.” (P20)
	Strategic systemic uncertainties	“There is no single, unambiguous strategy at the regional level… there’s no prospective strategy.” (P5) “There are entire departments that do not know what goals they have to go achieve, that they have to, let’s say, pursue, because maybe meetings have not been held, have not been explained.” (P19)
	Inadequate role recognition	“While on the medical side there is a perceived difference between the figure of the medical executive who makes certain decisions at a high level, on our side that is missing, that is, the influence we can have on provided care.” (P18)

Exolevel	Lacks in tailored education and professional development opportunities	“Courses offered at the local level… Do not always respond to my interest.” (P11) “Courses for coordinators have not been organized.” (P19)
	Lack of independence in decision‐making	“Still don’t completely have the autonomy to decide… because they still want to interfere.” (P18) “But here it’s difficult precisely because the departments are very large and then because the role is not yet structured in such a way that these department coordinators can be made operational, because they, in order to be able to perform this kind of role, should have the dashboards of the whole department in their hands.” (P17)

Mesolevel	Lack of structural and organizational support	“A coordinator… needs to be supported.” (P17) “The other problem is that I think there is a lack of supports, organizational supports.” (P20)
	Lack of coordination	“There is no interaction between any of us. Each coordinator does it his own way. You don’t know what happens with one or the other.” (P3) “Meetings… are organized, not as frequently as I would consider appropriate… many issues, in my opinion, should be handled as soon as possible.” (P22)

Microlevel	Ambiguities in the role expectations	“It’s as if the coordinator is perceived more as the manager… acting just as a liaison, being an intermediate figure, perhaps one would expect… that he would also be closer to those on the front line.” (P22) “Then if you want you can ask me what activities are not consistent with my coordinating role and I do anyway.” (P7)
	Bureaucratic burden	“As for the bureaucratic‐administrative dimension, I’ll tell you that actually what is missed is due to the fact that this dimension is just a lot, it’s too much and it’s often not consistent with the goals it’s designed for.” (P13) “Perhaps because we are so overburdened with bureaucratic activities, we have to answer to our management, we have to update certain things.” (P17)
	Lack of team cohesion and loneliness	“Perhaps there is not even a willingness to share and face… some issues that are related precisely to the actual loneliness of the managerial role.” (P6) “You can have super professionals and still have a department that is not working… because the team is missing.” (P8)
	Disjointed information flows	“The fact that we know more and more have different access points to take information… this thing here is not very clear and so there is not a clear overall project.” (P20) “We have little benchmarking with others, with other coordinators within the department, with other coordinators within the company, so we also learn little of others.” (P20)

Nurse manager level	Chronic time scarcity and priority skills	“Because I don’t have the time. 11 h a day and then I’m exhausted.” (P1) “They omit or delay activities because they lack time. So whenever there’s an increase in demands, they have to establish priorities.” (P20)
	Lack of experience	“Now it is different since I have managers who are 29… Very good… but lacking experience.” (P21)” “This means an absolute inability…poor perception, I would even say inability, to ensure the role, which is instead to govern resources and to understand that something like this then causes you cascading failures… it’s hard. Let’s say that the perception that we have, that has is that people have kind of improvised to perform this role of coordinators and they don’t always have the perception of what are the consequences of their missed actions.” (P19)
	Attitudes	“Meaning that everyone tends to only care about their business without actually caring about what’s going on elsewhere.” (P19)
	Sense of disengagement	“Perhaps a slight disaffection in a system that is putting a strain on you.” (P3)

*Note:* Legend: *P*: participant.

At the exolevel, participants reported a “*Lacks in tailored training and professional development opportunities*” for NMs, which fails to adequately support their ongoing professional growth. This does not mean that educational activities are not offered, but rather that they are not aligned with the expected needs (“*Courses offered at the local level*… *Do not always respond to my interest.*”, P11). Given the dynamic nature of the role, continuous tailored training is required to meet performance expectations. Furthermore, participants reported a “*Lack of independence in decision-making*” afforded to NMs, largely attributed to insufficient training, poorly defined role expectations and autonomy, or continuous interference from physicians (“*Still do not completely have the autonomy to decide*… *because they still want to interfere*”, P18). This limitation restricts their ability to establish clear priorities and decide which actions to perform.

At the mesolevel, participants reported the “*Lack of structural and organisational support*” revealing a perception of poor support from senior managers (“…*meeting once a month*…”, P17), while a lack of coordination was also mentioned, as NMs worked alone and missed opportunities to share problems and decisions within the group of managers (“*There is no interaction between any of us. Each coordinator does it his own way. You do not know what happens with one or the other*.”, P3).

At the microlevel, “*Ambiguities in role expectations*” suggest that NMs were often unclear about their responsibilities and the expectations within their units, particularly when balancing clinical and managerial tasks, resulting in activities that were not “*consistent with their coordinating role*” (P7). They also reported a “*Bureaucratic burden*,” i.e., numerous documentation and administrative tasks (P17) that take up time which could otherwise be spent on higher‐value managerial functions. In addition, participants described a “*Lack of team cohesion and a feeling of loneliness*”, leading to difficulties in delegating and sharing responsibilities with team members, which in turn further overburdens NMs. Finally, “*Disjointed information*” was cited as a cause of extra work: NMs often have to obtain missing information, verify data, check reports, and monitor the performance of their staff, which takes time and allows them to “*learn little about others*” (P20).

Some antecedents were also identified at an individual level. “*Chronic time scarcity*” forced NMs to complete only urgent or daily tasks, often at the expense of several other functions (“*Because I do not have the time. 11 h a day and then I exhausted*.”, P1). In addition, “Lack of experience” also made effective prioritisation difficult and increased the risk of essential management functions being overlooked (“*People have kind of improvised to perform this role of NMs*”, P19). Moreover, individual “*Attitudes*”—such as favouring certain aspects of the role over others—were reported to influence managerial performance (P19). A growing “*Sense of disengagement*” was also identified as a barrier to performing all expected NM functions (“*Perhaps a slight disaffection in a system that is putting a strain on you*.”, P3).

### 3.4. Consequences

Seven main consequences emerged across four levels (Table 5):

- At the NM level, effects were identified in two main domains: “*Emotional*” and “*Leadership issues*”. Emotional consequences included feelings of isolation, alienation, low energy, stress, disengagement, and frustration. In terms of leadership, participants reported an erosion of credibility, reduced authority and a perceived loss of openness and innovation.
- At the nursing staff level, participants reported “*Emotional*”, “*Communication*” and “*Professional issues*”. Emotional consequences included stress, discomfort, reduced mutual respect, frustration and demotivation among nurses. Failures in NM functions were also associated with poor information sharing, leading to confusion and misunderstandings. From a professional perspective, participants mentioned a lack of development opportunities and a deterioration in professional standards.
- At the unit level, “*Performance issues*” such as limited innovation or organisational development, a unit merely surviving and increased staff turnover were reported.
- At the patient and family level, “*Patient care issues*”, including a decline in the quality of care and the gradual erosion of patient‐centredness, were reported as consequences.

**TABLE 5:** Missed Nursing Management functions: levels, consequences and quotes.

Level	Consequences	Quotes
Nurse manager	Emotional issues ⁃ Loneliness ⁃ Alienation ⁃ Lack of energy ⁃ Stress ⁃ Dissatisfaction ⁃ Frustration	“So, this means that you will be working alone in the end.” (P3) “But there is dissatisfaction…” (P3) “Consequences I identify in me as a professional is to alienate myself a little bit too much…” (P9) “You start lacking energy… then you feel like you’re the only one pulling the strings.” (P10) “But of my own, then it depends on how one experiences it, as for me I feel that I am lagging behind and I know that there is something wrong, so, let’s say, I could identify the consequences for me, in terms of stress undoubtedly…” (P10) “Leaving out strategic activities, in my opinion, has resulted in a feeling of frustration…” (P15) “Then on the one hand there is the person with an organizational role, so he is stressed, dissatisfied and frustrated, because he thinks the company is asking him too much, and it’s often like that, but you also need to have the ability to state that it cannot always be like that, he thinks the company is asking him to solve problems and that’s it.” (P21)
	Leadership issues ⁃ Losing credibility ⁃ Losing authority ⁃ Losing the capacity to be innovative/being obtuse	“If there is a coordinator who doesn’t always manage to do everything, the effect is what is written in the books, you lose some credibility. You lose authority.” (P2) “And so, innovation, both in terms of technology, but also simply in terms of content, so choosing to be able to devote yourself to some content that is also a little bit different than the previous years and then also keeping yourself up to date, innovative and pioneering.” (P7) “Certainly, training allows you to open your horizons a little bit, compare yourself with others, find new strategies and new tools. The risk is a little bit of get stuck on what you’ve always done, how you’ve always done it, to also having a very…very obtuse, very limited point of view.” (P11)

Nursing team work	Emotional issues ⁃ Stress, discomfort ⁃ Lack of respect ⁃ Tension, conflicts ⁃ Frustration ⁃ Demotivation	“But it often happens that not that there is no proper respect for a person, sometimes there is no respect for the work of others, which means mine is always more important.” (P2) “I’ll give you an example, which is something I have experienced recently, the very fact of having many young colleagues who are inexperienced brings tension within the group, and this is felt at all levels, you know. However, the ability to relate is also to share and figure out how the best way to handle the situation.” (P4) “Let’s say, from the organizational point of view … but everything you do increases stress, discomfort, various issues that, then, indirectly, are reflected in the risk of errors, mistakes and working stress of the employee, the professional.” (P14) “This also caused frustrations within the group because there were misunderstandings.” (P15) “On the other hand, it leads to demotivation of the staff as well because the staff that are coordinated, or somehow related, need stimulation, especially in these years, in this era, in this historical period.” (P21) “We then moved on to intra‐staff conflicts because of course then dynamics of lack of clarity are generated, or a somewhat chaotic management, so among the staff over time conflicting situations have been generated. To date we have, instead, a staff versus coordinator conflict basically.” (P22)
	Communication issues ⁃ Lack of knowledge and information ⁃ Confusion ⁃ Misunderstandings	“In short, when working, you shouldn’t be done something that has not been shared…” (P2) “Lack of knowledge, because if you don’t know that we said something at a meeting and I also put out a minute, but if you haven’t read it, meanwhile so much time passes before this knowledge reaches you.” (P3) “So, you’re not the one giving the answers and that sometimes creates conflict, or it creates disparity of information, confusion, that is true.” (P13) “So, if I postpone strategic activities… it means that I postpone communication for 24 h.” (P15) “Misunderstandings within the group.” (P15) “In my opinion, some things are not addressed, not discussed openly by nurses with their managers, because if they’re not feeling them close, not perceiving their role and importance within the organization, they’ll say “even if I tell him, nothing changes,” you know? In my opinion, that is the consequence.” (P18) “There are entire departments that do not know what goals they have to go achieve…” (P19) “This creates problems in collective bargaining, which they don’t even perceive.” (P19)
	Professional issues ⁃ Lack of professional development ⁃ Decline in the professional quality ⁃ Lack of motivation	“You let the profession die.” (P3) “We only do minimum welfare, so there’s the risk of bringing down the quality of profession.” (P3) “The consequences that we certainly have is that you create a team but then if they don’t work well and are unmotivated, people apply for transfer and you have lost those skills. The moment you start to lose those skills and you end up with a team that doesn’t work in synergy and doesn’t work in the way you expect, it’s a whole cascade that is generated, both in care outcomes and in professional satisfaction and growth that is not there. So those are the consequences. Then relationships improve, relationships improve in every sense because of course there is also an aspect of satisfaction. The fact of having and seeing care outcomes, of seeing that the work you do brings positive health outcomes for the person, seeing that the nurse’s care activity makes a difference in the care process is motivating.” (P17) “I always focus on the working group, because I have noticed over the past 3 years, the moment the group inside has dynamics that work and that push toward a certain type of care, that kind of care is delivered, let’s say, at very high levels. The moment you have groups where there is a high turnover, there is so little communication, there are so many, let’s say, difficult dynamics, where you are not maybe on the same…then, everybody can have different ideas, but I mean that we have to share an idea of what for us is the best care we can give to the patient. When that happens within a group, a group works well and the standard of care is high.” (P18)

Unit	Performance issues ⁃ Lack of innovation and organizational development ⁃ Increased turn over	“Well, I am a great supporter of teamwork. In my opinion, we can’t go anywhere if in any environment we don’t form a team…” (P1) “But I have seen in my work that people need to see the coordinator as well, he has to check if people are satisfied or if they don’t feel satisfied, if they can create problems for the organization.” (P2) “…then you pay for it in bringing projects forward.” (P3) “And then you don’t let the group grow, you don’t let the group grow here, you don’t let the group of association grow.” (P3) “There are wards people run away from. They’ll go somewhere else and, therefore, are lost.” (P19)

Patient/family	Patient care issues ⁃ Poor quality of care ⁃ Missing patient‐centered care	“In the sense that we miss, we miss what is our focus, which for me is the patient, that’s it.” (P12) “Let’s say, from the organizational point of view you risk less directly, in the short term, with respect to patients themselves…” (P14) “By actually delaying certain activities… we are probably indirectly still going to affect the patient…” (P16) “The consequences of the coordinator… impact a lot on the quality of care…” (P17)

*Note:* *P*: participant.

## 4. Discussion

As far as we know, this is the first study to examine which functions and activities are missed or postponed by NMs, along with the related reasons and consequences. We explored the phenomenon from both insider and outsider perspectives, including clinical nurses supervised by NMs, nurse executives with broader system‐level supervisory roles, nurse educators from academic institutions, and representatives of professional associations reflecting collective issues in the daily implementation of the expected role. Participants represented a range of professional experiences, roles, formative generations [50], and levels of experience, from novices (with 1 year’s experience) to experts, providing a rich and diverse range of perspectives. Overall, NMs also miss or postpone functions, suggesting that, as with clinical nurses, nurse educators and more recently physicians, missing or postponing is a widespread phenomenon.

### 4.1. Missed Nursing Management Functions and Activities

The dimensions identified as “missed” refer to functions rather than specific activities. Participants described NMs as responsible for processes rather than individual tasks, clearly distinguishing the phenomenon of missed care in the clinical domain from that in the management domain [2]. Furthermore, while the literature on MNC describes a wide range of missed tasks—including technical, cognitive and emotional ones—only a limited number of missed functions have emerged among NMs. Overall, these missed functions are more clearly defined and homogeneous than the variety of missed care activities found among clinical nurses in the context of unfinished nursing care research.

Four main functions have been identified as being missed. The first involves planning and monitoring, which are core functions of NMs. They are expected to articulate a vision aligned with strategic goals and to oversee its practical implementation [20]. Participants appear to rely on day‐to‐day management, struggling to set goals, define priorities and monitor progress [20]. This reactive management approach, focused on daily issues, is seen as particularly problematic as it does not align the entire unit with the expectations of the healthcare system, slows its development, and undermines the perception of the coherence of its mission.

The second function involves the lack of not only physical but also emotional, intellectual and social presence within the unit. This absence reflects a detachment from the place where care is provided, leading to a form of remote management, detached from daily contact, visibility and proximity. Participants emphasised the absence of a manager perceived as “*one of us*”—someone who supports the team, cares for individual nurses and remains attentive to patients. This “missing” presence signifies not only an inability to be truly present but also difficulty in understanding the real healthcare setting, creating a distance that fragments management and puts NMs at risk of becoming disconnected from the clinical context. This can affect both the quality of care and the working environment.

The third function concerns coordination activities closely related to the NM role [20]. Firstly, the amount of information they receive is perceived as unmanageable. This may be related to difficulties in dealing with digital tools and hospital information systems, which are often fragmented and lack interoperability [51]. As a result, the flow of information is not managed effectively, and information sharing—an essential component of leadership involving presence, dialogue and consensus building—is significantly weakened. Delegation of tasks, if not properly prepared, supervised or followed up, can result in decentralised activities gradually becoming the responsibility of clinical nursing staff, who are already overloaded with numerous tasks. This contributes to nurses feeling burdened with tasks that are not their responsibility; they may feel recognised or valued as they attempt to take on a different role, such as that of a manager. Finally, NMs’ ability to be part of a coordinated management team and to work horizontally with colleagues is also missed. This highlights not only a weakness in the management of individual operational units, but also a wider challenge: the weakening of integration across different units. This may result in a lack of oversight and control, leading to a perceived loss of direction and cohesion in overall management.

The fourth missed function concerns the perceived lack of development at multiple levels—the department, nursing staff, individual nurses, students and even the NMs themselves. This perception adds another dimension: the feeling of being not only out of context, but also out of time. If NMs are not able to continuously develop themselves and their staff, they risk proposing outdated solutions, interventions and strategies that no longer meet current challenges.

### 4.2. Antecedents

Overall, as with MNC [8, 52], the reasons for Missed Nursing Management may be found at multiple levels—not only within the unit but also at higher system levels—suggesting that a system‐wide rather than a siloed approach is required. Moreover, some antecedents can be addressed at the hospital level, while others require a broader, system‐wide approach. Similarly, certain antecedents call for organisational strategies, whereas others demand individual investment and engagement.

The strain of the nursing shortage and the ongoing postpandemic recovery period are affecting the ability of NMs to fulfil their expected functions. They worked hard during the pandemic and continue to face the daily challenge of ensuring adequate staffing for shift work despite the staff shortage [53]. The daily provision of expected care through constant adjustment of shift schedules—which are always completed and not reported as missing activities—requires considerable time and energy, often at the expense of other functions that are then neglected. The low recognition of the NM role at a systemic level contributes to a lack of awareness of management functions, suggesting that their visibility and recognition need to be increased so that available energy can be channelled into fulfilling core responsibilities rather than constantly justifying the role itself.

Educational and professional organisations should also implement strategies to address antecedents identified at the exolevel. Professional development for NMs should be designed to strengthen their decision‐making and prioritisation skills. While an entry‐level qualification (e.g., a master’s degree) is usually required to access the NM role, there is a lack of clearly defined continuous educational strategies for NMs, in contrast to the more structured pathways available for clinical nurses [54].

At the mesolevel, structural and organisational support and coordination remain underdeveloped, suggesting that NMs leading multiple departments should encourage consistent and coherent support throughout the management hierarchy. Lack of organisational support is also a recognised reason for poor quality of care provided by clinical nurses, indicating that these deficiencies may have a dual impact on both MNC and managerial functions.

At the unit level, role ambiguity—particularly the tension between clinical and managerial roles—is a key issue [55]. While the dual role is seen as beneficial in some contexts, in others it is perceived as an obstacle because integrating the two areas proves difficult [11]. The bureaucratic burden, weak team cohesion, and disjointed flow of information suggest that the system needs to be reorganised at the unit level.

Finally, the individual characteristics of NMs are similar to those documented for clinical nurses. Prioritising management tasks requires different skills from those needed for prioritising clinical tasks and related experiences. Therefore, support systems should be strengthened, especially for those transitioning from clinical to management roles. Some attitudes, such as excessive individualism—which is also mentioned in the literature on missed care—can contribute to missed management tasks [56]. Similarly, disengagement can be both a cause and a consequence of missed management activities.

### 4.3. Consequences

All identified consequences are located at the microsystem level; there is no direct evidence of an influence on higher system levels as has been documented for MNC [57]. The impacts seem to remain internal within the professional nursing system and affect nursing management, its staff and organisational performance, with some traces of external impact on patients and families. These impacts appear to follow a cascading dynamic: lower motivation in NMs or limited organisational development and innovation, for example, reduce the attractiveness of the profession for nurses and hinder their professional development. This, in turn, can impact the NM’s experience and further weaken their leadership skills. The potential effect described by participants suggests an innovative perspective through which to interpret the well‐documented challenges in nursing practice (e.g., turnover and dissatisfaction), where the critical role of leadership is well emphasised [58]. This perspective is not just about the presence or absence of leadership qualities but rather the inability to fully express or sustain the nursing staff, also in policies [59], as seen in the expression of Missed Nursing Management functions and ultimately the risk of losing them (both NMs and nurses) altogether.

### 4.4. Strengths and Limitations

This study has several strengths and limitations. First, it involved only nurses: on the one hand, this is a strength as it ensures focus on a single profession; on the other hand, it provides only an internal perspective of the profession, suggesting the need for a broader perspective in future studies. Moreover, the study includes nurses working in diverse units: while this may increase the richness of the data, it may also introduce the risk of overgeneralisation, reducing the likelihood of specific, actionable findings. Furthermore, experiences were collected freely and without a predefined structure regarding the functions and activities expected of NMs, thus avoiding a rigid framework and not assessing daily functions enacted, which may also depend on the context. This methodological decision was intentional to allow freedom of expression and to acknowledge that the role of NMs is still not clearly defined [20, 40, 41] and may be shaped by contextual needs. Future studies should build on this knowledge to refine the conceptualisation of the phenomenon by detecting missed function/activities at a more granular level.

## 5. Conclusions

Essential functions expected of NMs are missed, including critical components of the management cycle such as planning, supervision, presence and visibility alongside nurses, coordination, ongoing adaptation and professional development. While NMs miss some functions, others, such as resource management or problem solving, remain part of their daily routine, as they are urgent and essential for the unit’s daily survival. Antecedents of missed nursing management functions and activities can be found at multiple levels, including individual NM and the micro‐, meso‐, exo‐ and macrosystems. This suggests that a comprehensive, system‐wide approach is needed rather than isolated or siloed interventions. The consequences of missed nursing management functions and activities are primarily at the microsystem level, affecting NMs themselves, their nursing teams, organisational performance and potentially the quality of care for patients and families.

### 5.1. Implication for the NM

Overall, NMs appear to practise a reactive and immediate form of management, in which more strategic functions—such as long‐term planning, staff development and a visible and supportive presence—are often overlooked or deprioritised. Further research is required to determine whether this phenomenon is consistent across different contexts or shaped by specific cultural or organisational factors. Some root causes can be addressed at the hospital or departmental level, while others may require action at a broader policy or public health level. Similarly, certain problems may require organisational strategies, while others call for individual commitment and professional development. Framing the findings as modifiable managerial practices, rather than solely individual shortcomings, can inform policies that strengthen managerial capacity, promote accountability and ultimately enhance both work environments and care quality. Leadership emerges as a strategic component in ensuring staff well‐being and promoting organisational development and performance. However, the results of this study suggest a possible new direction for research: perhaps it is not a lack of leadership competencies, but rather a lack of opportunities to exercise them [60]. NMs are often unable to fulfil the core functions of effective, supportive leadership—not because of a lack of competence, but due to strategic, structural and contextual constraints. It is therefore critical to develop strategies that not only enhance leadership capabilities but also enable the full realisation of leadership functions, creating the conditions for NMs to effectively fulfil their strategic and supportive roles.

Moreover, enhancing systemic recognition of the NM role, investing in structured and regular opportunities for professional development, clarifying roles at the unit level and strengthening organisational support mechanisms may help protect managerial time and energy, enabling NMs to focus on core functions and activities. In addition, the findings suggest the importance of early and targeted interventions aimed at supporting NMs at the unit level to preserve their managerial effectiveness.

## Author Contributions

Conceptualization: AP, SC and MF. Data curation: All authors. Formal Analysis: SC, GTE, MF and AP. Funding acquisition and investigation: SC, GTE, MF and AP. Methodology: SC, GTE, MF and AP. Project administration: AP. Resources, software and supervision: AP, SC and GTE. Validation: SC, GTE, MF and AP. Visualization: SC, GTE, MF and AP. Writing–original draft: SC, GTE, MF and AP. Writing–review and editing: All authors.

## Funding

This study did not receive any grants from funding agencies like government, private or nonprofit organizations.

Open access publishing facilitated by Universita degli Studi di Udine, as part of the Wiley ‐ CRUI‐CARE agreement.

## Conflicts of Interest

The authors declare no conflicts of interest.

## Supporting Information

Additional supporting information can be found online in the Supporting Information section.

## Supporting information


**Supporting Information 1** Supporting Table 1. COnsolidated criteria for REporting Qualitative research Checklist.

**Supporting Information 2** Supporting Table 2. Strategies to promote rigour.

**Supporting Information 3** Supporting Table 3. Missed Nursing Management Functions, Activities and Quotes.

**Supporting Information 4** Supporting Table 4. Missed Nursing Management antecedents according to their levels and quotes.

## Data Availability

The data that support the findings of this study are available in the Supporting Information of this article.
