# Supplementary material for: Functions and Activities Missed by Nurse Managers, Their Antecedents and Consequences: Findings From a Qualitative Study
Source: J Nurs Manag. 2026 Jun 29;2026:4992301. doi: 10.1155/jonm/4992301 (PMC13311724; doi:10.1155/jonm/4992301)
Supplement: Supplementary file 1 — Supporting Information 1 Supporting Table 1. COnsolidated criteria for REporting Qualitative research Checklist. [file JONM-2026-4992301-s001.docx]

**Supplementary Table 1.** COnsolidated criteria for REporting Qualitative research Checklist [1]

| **Topic** | **Item No.** | **Guide Questions/Description** | **Briefly description** |
| --- | --- | --- | --- |
| **Domain 1: Research team and reflexivity** | | |  |
| *Personal characteristics* | | |  |
| Interviewer/facilitator | 1 | Which author/s conducted the interview or focus group? | MF and SC |
| Credentials | 2 | What were the researcher’s credentials? E.g. PhD, MD | MNS; PhD |
| Occupation | 3 | What was their occupation at the time of the study? | Student; Research fellow |
| Gender | 4 | Was the researcher male or female? | Both female |
| Experience and training | 5 | What experience or training did the researcher have? | (SC) was educated at the PhD level and conducted several qualitative studies  (MF) was a Master student |
| *Relationship with participants* | | |  |
| Relationship established | 6 | Was a relationship established prior to study commencement? | None |
| Participant knowledge of the interviewer | 7 | What did the participants know about the researcher? e.g. personal goals, reasons for doing the research | The participants were informed only regarding the aim of the study |
| Interviewer characteristics | 8 | What characteristics were reported about the inter viewer/facilitator? e.g. Bias, assumptions, reasons and interests in the research topic | The participants were informed regarding the research interests of the interviewers |
| **Domain 2: Study design** | | |  |
| *Theoretical framework* | | |  |
| Methodological orientation and Theory | 9 | What methodological orientation was stated to underpin the study? e.g., grounded theory, discourse analysis, ethnography, phenomenology, content analysis | Qualitative study, based on inductive and deductive thematic analysis |
| *Participant selection* | | |  |
| Sampling | 10 | How were participants selected? e.g. purposive, convenience, consecutive, snowball | Participants were identified purposefully |
| Method of approach | 11 | How were participants approached? e.g. face-to-face, telephone, mail, email | The senior research (AP) contacted the potential participant in advance via email; no refused the invitation |
| Sample size | 12 | How many participants were in the study? | 22 in total |
| Non-participation | 13 | How many people refused to participate or dropped out? Reasons? | None |
| *Setting* | | |  |
| Setting of data collection | 14 | Where was the data collected? e.g., home, clinic, workplace | The data collection was performed in the place decided with the participants according to their preferences |
| Presence of non-participants | 15 | Was anyone else present besides the participants and researchers? | Only the researchers (SC, MF) |
| Description of sample | 16 | What are the important characteristics of the sample? e.g. demographic data, date | A few data have been collected in order to protect anonymity  Table 2 |
| *Data collection* | | |  |
| Interview guide | 17 | Were questions, prompts, guides provided by the authors? Was it pilot tested? | The interview guide was established a priority (Table 1) and piloted |
| Repeat interviews | 18 | Were repeat inter views carried out? If yes, how many? | No, the interviews were not repeated |
| Audio/visual recording | 19 | Did the research use audio or visual recording to collect the data? | The interviews were audio recorded and transcribed verbatim |
| Field notes | 20 | Were field notes made during and/or after the interview or focus group? | Yes, and discussed among researchers |
| Duration | 21 | What was the duration of the inter views or focus group? | Around one hour |
| Data saturation | 22 | Was data saturation discussed? | Yes, by all researchers involved in the process of data collection (AP, SC, MF) |
| Transcripts returned | 23 | Were transcripts returned to participants for comment and/or correction? | No |
| **Domain 3: analysis and findings** | | | |
| *Data analysis* | | | |
| Number of data coders | 24 | How many data coders coded the data? | Three (MF, SC, AP); then, transcripts have been translated and a fourth researcher have been involved in the data coding (GTE) |
| Description of the coding tree | 25 | Did authors provide a description of the coding tree? | Yes, the coding tree were described in Supplementary Table 3, 4 and in Table 5 |
| Derivation of themes | 26 | Were themes identified in advance or derived from the data? | The themes have been derived from the data |
| Software | 27 | What software, if applicable, was used to manage the data? | None |
| Participant checking | 28 | Did participants provide feedback on the findings? | Participants did not provide feedback on the findings |
| *Reporting* | | | |
| Quotations presented | 29 | Were participant quotations presented to illustrate the themes/findings? Was each quotation identified? e.g. participant number | Quotations have been extracted and presented in an anonymized fashion |
| Data and findings consistent | 30 | Was there consistency between the data presented and the findings? | Yes, the findings derived directly from the data through an inductive process |
| Clarity of major themes | 31 | Were major themes clearly presented in the findings? | Major themes are clearly described |
| Clarity of minor themes | 32 | Is there a description of diverse cases or discussion of minor themes? | Minor themes are also described |

**Legend:** MNS, Master of Nursing Science; PhD, Philosophy Doctor; Initials on bracket, see authors.

1. A. Tong, P. Sainsbury and J. Craig, “Consolidated criteria for reporting qualitative research (COREQ): a 32-item checklist for interviews and focus groups,” *International journal for quality in health care: journal of the International Society for Quality in Health Care* 19, no. 6 (2007): 349–357, <https://doi.org/10.1093/intqhc/mzm042>.
