# Supplementary material for: Functions and Activities Missed by Nurse Managers, Their Antecedents and Consequences: Findings From a Qualitative Study
Source: J Nurs Manag. 2026 Jun 29;2026:4992301. doi: 10.1155/jonm/4992301 (PMC13311724; doi:10.1155/jonm/4992301)
Supplement: Supplementary file 2 — Supporting Information 2 Supporting Table 2. Strategies to promote rigour. [file JONM-2026-4992301-s002.docx]

**Supplementary Table 2.** Strategies to promote rigor [1]

| - ***Credibility:*** a purposeful sample of nurses in different roles was involved, thus providing varied perspectives on the phenomenon. The interviews were transcribed verbatim immediately. Both senior and junior researchers, including those at the international level, participated, as well as a nurse manager (MF) to ensure an insider perspective. Additionally, individual assumptions of data analysts were shared among researchers before data analysis. - ***Dependability:*** the interview questions were identified in a preliminary manner and used consistently across participants; data analysis was conducted according to the research questions established in the research protocol. Data analysis was carried out by a group of researchers and experts in the field. - ***Confirmability:*** quotes were extracted from the interviews and provided in detail, while preserving anonymity. An audit trail extract is provided as Supplementary Table 3 and Supplementary Table 4. - ***Transferability:*** the research context has been described (see methods section). |
| --- |

1. L. Maher and G. Dertadian, “Qualitative research,” *Addiction (Abingdon, England)* 113, no. 1 (2018): 167–172, <https://doi.org/10.1111/add.13931>.
