# Supplementary material for: Functions and Activities Missed by Nurse Managers, Their Antecedents and Consequences: Findings From a Qualitative Study
Source: J Nurs Manag. 2026 Jun 29;2026:4992301. doi: 10.1155/jonm/4992301 (PMC13311724; doi:10.1155/jonm/4992301)
Supplement: Supplementary file 3 — Supporting Information 3 Supporting Table 3. Missed Nursing Management Functions, Activities and Quotes. [file JONM-2026-4992301-s003.docx]

**Supplementary Table 3.** Missed Nursing Management Functions, Activities and Quotes

| ***Themes: Functions*** | ***Sub-themes: Activities*** | ***Quotes: Examples*** |
| --- | --- | --- |
| ***Missing systemic planning and monitoring*** | **Missing setting unit goals and priorities to ensure that the strategic mission is addressed** | *“Having a management that tells you ‘This is the project and we are working for it, communicates that and clears away the doubts... would be more accepted by the employees than working without knowing anything.” (P15)*  *“...corporate goals... how they are decided... how they are assigned to nurses...” (P18)*  *“...creating a working group... deciding the roles... omitted care...” (P18)*  *“Middle management roles… can’t do any planning… they can’t have clean, critical thinking with respect to higher goals.” (P21)* |
|  | **Missing using data and indicators** | *“Yes, so, in my opinion… the review of activities… unfortunately doesn't get enough attention.” (P13)*  *“There is a big gap on data management.” (P15)*  *“We never measure care outcomes… because the opportunities are related to corporate projects.” (P17)* |
| ***Missing effective presence in the clinical environment*** | **Missing being present, visible and ensuring proximity** | *“I really miss this contact that I have with a world that I define as trench. Because things have to be changed there. And if you are there you also give a shake, an encouragement to those who are in the trenches at the moment.” (P1)*  *“But I belong to the world of my co-workers first... I understand on the fly how the organization of that department works. And I really miss that contact.” (P1)*  *“Coordinators no longer have ownership of their own working time.” (P2)*  *“It seems to me that it is a setback...” (P2)*  *“...missing in my context is perhaps the presence of the coordinator in the care setting...” (P16)*  *“...willingness to do a shift change, being readily available... fairness in giving evaluations...” (P16)*  *“By not practicing clinical care… we don't even know what path the patient takes.” (P17)*  *“Handovers… have to be supervised… the coordinator works with the group.” (P17)*  *“As a coordinator, it is important to also be very present in the context of clinical care… however sometimes maybe we are not present in the ward." (P17)*  *“By not practicing clinical care… we don't even know what path the patient takes.” (P17)*  *“Definitely the nursing management... lacks in being present...” (P17)*  *“Leadership should also periodically go to the wards...” (P17)*  *“As a coordinator, it is important to also be very present in the context of clinical care… however sometimes maybe we are not present in the ward.” (P17)*  *“I think the thing that is often overlooked, because I have seen this being done a lot by coordinators, is to be present during these steps. The coordinator has to lead and has to make sure that the whole team goes in, is, let's say, coordinated, meaning that maybe, I'm not saying to suggest what to do, however maybe have moments of...for example the case manager nurse me...we had...excuse me, the continuity nurse, not the case manager, the continuity nurse worked in my office, the adjoining one, however we were daily defining and figuring out how we were proceeding with the discharges and especially figuring out what services to activate.” (P17)*  *“...you don't feel the presence of our health care leadership at our level...” (P18)*  *“...even as a physical place... she never comes to the ward...” (P18)*  *“...coordinator is someone you can interact with often.” (P18)*  *“The coordinator today takes refuge behind computers… but the coordinator, he actually has to be in the department, at least for a great percentage of his time.” (P19)*  *“There are departments people run away from because they are not properly managed... a work environment is created that is sometimes even harassing.” (P19)*  *“The question is, ‘but where is management?’ That’s what those who are then underneath think and it’s not nice.” (P22)* |
|  | **Missing taking care of my team** | *“So also, to take care of people of work groups.” (P6)*  *“Another aspect that needs to be managed is also having a relationship with the staff i.e., you have to know the staff. You have to know the issues that they have, because the moment I start a shift, I have to know who I can call and maybe put in difficulty when calling. Then it's logical, the priority is to ensure patient safety and to ensure the delivery of services, but maybe if I know my staff well, maybe I can also manage the organization of the shift schedule in such a way that I put people in less difficulty and that makes them more loyal to their work.” (P17)*  *“Even from the point of view of the employee’s well-being… it would be useful; it would be nice… to approach the needs of the professionals.” (P22)* |
|  | **Missing taking care of individual emotional and relational needs** | *“...it's the face-to-face relationships that matter most...” (P1)*  *“I also miss the opportunity to converse with the newly-hired.” (P1)*  *“I have lost sight of them a little bit...” (P12)*  *“...direct relationship is missed.” (P13)*  *“I regret not being able to give a personal response.” (P13)*  *“...putting off often... meetings with employees in fragility.” (P15)*  *“...willingness to do a shift change, being readily available... fairness in giving evaluations...” (P16)*  *“...the coordinator has to lead... not to suggest what to do, but maybe have moments of coordination...” (P17)*  *“...know their staff well... up-to-date file of the person...” (P17)*  *“...you have to know the issues that they have... manage shift schedules accordingly...” (P17)*  *“A worker who is serene, is motivated… works more effectively and efficiently, and this is often, in my opinion, forgotten.” (P22)* |
|  | **Missing seeing patients** | *“Instead, you tend to neglect them more and more… I am not able to see the patients for days… this is something that makes me sick.” (P4)* |
| ***Missing coordination and continuous alignment to the expected goals*** | **Missing managing information flows and deadlines** | *“...delayed care, omitted by the coordinator... when the clinical nurses do not receive some information in a timely manner...” (P16)*  *“...adequate passage of information... in the timeframe that is useful...” (P16)*  *“So, if you can curb that little daily drama every time you have something new within the department, however, you have to share it first, you have to have the professionals on your side, in my opinion. That is one of the things that is omitted, right now eh, where I have been working, these last two, three years.” (P18)* |
|  | **Missing communicating and sharing information** | *“I lack contact with these figures...” (P1)*  *“There is no time... to exchange ideas...” (P2)*  *“But it's also unfair to deal with a problem after one has thought about it just for a moment...to deal with people with whom, let's say, one has to discuss an important problem.” (P2)*  *“That's why we need such moments to meet, which are not wasted time. If, on the other hand, the meeting is made to say: from tomorrow you have to do this and that, because I was told so... this is a mechanism that nobody would like. However, this is what we experience in our everyday life.” (P2)*  *“Impossibility to organize staff meetings...” (P4)*  *“Probably, thinking about the past, we had more structured moments to share the same issues, if I think of the companies, I have always worked, except for the last 8 years, in the territorial company, but in the hospital company there were systematic meetings to share the same issues.” (P6)*  *“There's a lack of sharing...” (P15)*  *“In practice, in my opinion, a delayed care, omitted for example by the role of the coordinator, which is the one closest to my context, arises, for example, when the collaborators, the clinical nurses do not receive some information in a timely manner, coming, for example, from the health professions leadership, information that should be given to us, starting with our nursing coordinator.” (P16)*  *“...meetings are not actually organized either with the coordinator or at the departmental level.” (P16)*  *“...staff meetings... a time of confrontation... a chance to explain...” (P17)*  *“...sharing decisions before I put you in an already established situation...” (P18)*  *“...sometimes lacking... the step of creating a working group...” (P18)*  *“...sharing decisions before I put you in an already established situation...” (P18)*  *“These priorities basically lead you to certain conditions, to do some things that you prioritize and to postpone, to delay or omit some other things that you have to do later, from reading the emails, to applying instructions or recommendations that are made by the various stakeholders which could be the medical leadership, the health professions leadership, myself, rather than the facility directors or it could also be simply the requests of their own staff.” (P20)* |
|  | **Missing supervising and controlling** | *“The emergency trolley… is an activity that should be under the control of the nursing coordinator and instead… is completely delegated.” (P16)*  *“Each department decides how to handle certain activities.” (P16)*  *“Handovers… have to be supervised… the coordinator works with the group.” (P17)*  *“We give rules and then everyone makes up their own rules within the departments.” (P19)*  *“The endurance, for example, of staff training. We know, when conducting checks with respect to mandatory staff training, that there are some areas that are not properly managed.” (P20)* |
|  | **Missing holding meetings with middle managers** | *“The thing I miss the most is the contact with the intermediate structures, let's call them that, which are the coordinators.” (P1)*  *“But I belong to the world of my co-workers first... I understand on the fly how the organization of that department works. And I really miss that contact.” (P1)*  *“I really miss this contact that I have with a world that I define as trench. Because things have to be changed there. And if you are there you also give a shake, an encouragement to those who are in the trenches at the moment.” (P1)*  *“I miss so much having contact with my coordinators...with the coordinators of the operating units, that is, the presence in the wards to touch what I see...” (P1)*  *“Coordinators no longer have ownership of their own working time.” (P2)*  *“Now for both the management part, middle management, but also top management, structured meetings are fewer, there are none at all.” (P6)*  *“I would like to deal more with the aspect of building trusting relationships with colleagues working in the various companies.” (P6)*  *“Organizational functions that are omitted within the system in which I work, what I perceive, both above me at the organizational hierarchical level and below, are the communication steps.” (P15)*  *“This obviously takes a lot of time, because everyone has their own vision and it is difficult to put together so many perspectives.” (P15)* |
| ***Missing to promote unit, staff and profession development*** | **Missing dedicating time to students** | *“I think it is difficult to find the space to cultivate more of a relationship with students.” (P9)*  *“I don't even know how to be part of the internship, together with the student.” (P9)*  *“We would probably need to find moments of listening. Or of, we are also thinking about this lately, of the student to somehow bring the world of the university closer to the world of work.” (P9)*  *“I can manage, both in terms of time, and in terms of energy, to take for example more time to conduct a mini-check at the patient's bedside, or discuss, assist, be part of the internship, but I don't even know how, together with the student.” (P9)*  *“What I tend to omit or suffer a little bit are those activities that detach me from the student.” (P10)*  *“I would gladly invest that hour and a half that I use for correction… at the patient’s bedside.” (P10)*  *“What I tend to omit, partly because I can't always recognize the usefulness of it all the way through, partly because it's an activity that you do that is detached from the student anyway, that of report correction. I mean that is something that I think I tend to postpone, to give, let’s say, that is secondary, that might actually...definitely has some value to the student's journey.” (P10)*  *“Even when it comes to training that one personally chooses, for example postgraduate courses or advanced courses, you know, it is not that I perceive a lot of support for a choice that, instead, is laudable, should be supported by a figure like the coordinator, who basically also has a coaching function, or could potentially be a mentor as well…” (P22)* |
|  | **Missing planning effective educational strategies and acting as a coach/mentor** | *“In the training role, maybe as a teacher, there could be a space dedicated to planning. So, a space for innovation.” (P7)*  *“So it's not just being a classroom lecturer, but embedding the activity within a much more global and systemic pathway. So probably being able to spend more time on planning, on identifying the resources needed to make effective workshops, on how to involve experienced professionals, rather than just on these aspects, would be functional in order to be able to try to ensure that we are offering something that is more and more in line with the needs of our students.” (P7)*  *“Another aspect that, let's say, I find that the coordinator maybe misses is what I was telling you earlier, to follow up on the staff introduction stages, because you can it to a mentor in whom you have utmost confidence, that's perfectly fine, however it's not like the newly hired nurse has to be constantly following a nurse all the time because that's his nurse, however it's certainly important for the coordinator to be proactive, to get informed, to go there, try to see, work, maybe with a trivial excuse, together with the newly hired person saying “come I'll explain this thing, let me see how you do this thing,” in such a way that you understand how much this person is in line with the directions because if I ask you “excuse me, will you book this thing for me, please” or I ask you “tell me how this patient is today and what have we done or what does he need” or go and listen to the handovers.” (P17)*  *“To understand, because you're going to have people who are good and people who are less good and, therefore, you need to understand who needs to grow, who doesn't need to grow, checking how the training of their employees is going, what they can do. The issue of competency profiles, right? That is, I have to know what each person can do, what they can’t do, and most importantly, if there is something they should know how to do but they don't actually know how, I have to intervene with some training and then check if their behaviour then aligns with the requirements.” (P19)*  *“So, that has been lacking in my experience, and I don't think the training needs are being adequately supported, in short. Well, everything comes from above but without any particular interaction between the coordinator and the professionals, so just from above, which may be at the corporate level, or at the regional one, so now for example radiation protection is the main goal, so that simply must be done, however with respect to individual courses and activities in which someone might be interested there is not much openness.” (P22)* |
|  | **Missing self-reflection and learning** | *“Meaning that one's training activity at this time is a bit missed, in the sense that you devote time to the organization, you devote time to your colleagues, you are focused on that and you have a bit of a hard time engaging in activities for yourself, for your professional growth, to enrich your skills. And so if all the legislation comes and you have to study it, everything that is related to your potential development instead becomes less important, because you are focused on the others than on your role anyway.” (P13)*  *“The fact of saying, “Let's keep up to date,” they keep up to date the moment a company procedure comes along and they have to comply with that, but the fact of independently studying a resolution in depth, of deepening a certain rule, a certain journal, a literature article, they struggle in doing so, they struggle a lot. Here I really understand it as missed care because there is a lack of that intellectual stimulus and professional updating that could enable a different management of even operational, group, organizational dynamics and so on.” (P21)* |

**Legend.** P, participant.
