# Supplementary material for: Functions and Activities Missed by Nurse Managers, Their Antecedents and Consequences: Findings From a Qualitative Study
Source: J Nurs Manag. 2026 Jun 29;2026:4992301. doi: 10.1155/jonm/4992301 (PMC13311724; doi:10.1155/jonm/4992301)
Supplement: Supplementary file 4 — Supporting Information 4 Supporting Table 4. Missed Nursing Management antecedents according to their levels and quotes. [file JONM-2026-4992301-s004.docx]

**Supplementary Table 4.** Missed Nursing Management antecedents according to their levels and quotes

| ***Level*** | ***Antecedents*** | ***Quotes*** |
| --- | --- | --- |
| ***Macro-level*** | Systemic challenges (shortages and post-pandemic recovery) | *“I have to tell you the truth, that right now the Order of ***** has difficulties, like many Orders nationwide, because of no vax issues, etc., that have a little bit sent everything that was ... They blew budgets, stuffs, so we are a little bit in trouble. So we're also a little bit stuck, because we do the minimum, not because we don't want to, but because they've blocked us, they're hindering us because they've stopped us from spending money.” (P3)*  *“Maybe COVID has created difficulties, maybe so much has been asked, maybe people are tired of earning little, so many causes.” (P3)*  *“Resources are not that much; you have to do without somewhere.” (P3)*  *“COVID has definitely resulted in a swing in all departments.” (P7)*  *“Due to problems of shortage of staff... we cannot always ensure the standards we should ensure.” (P9)*  *“The simplest reason might be the number of staff you have.” (P20)*  *“A situation whereby they live a little bit like the nurses who work in the clinic, in my opinion, with maybe not an adequate number of workers. An inadequate number compared to the size and complexity of the facilities.” (P20)* |
|  | Strategic systemic uncertainties | *“There is no single, unambiguous strategy at the regional level… there's no prospective strategy.” (P5)*  *“So what I cannot do, I cannot do because there is no political will to do it.” (P5)*  *“We are very focused on doing here and now... because probably complex systems lead you to overstructure.” (P6)*  *“It's a six-month hole where we don't have a certainty… we do it with common sense, but without a specific procedure.” (P15)*  *“…you live in the contingency of the day-to-day.” (P19)*  *“There are entire departments that do not know what goals they have to go achieve, that they have to, let's say, pursue, because maybe meetings have not been held, have not been explained.” (P19)*  *“They omit to read the regional resolutions carefully.” (P21)* |
|  | Inadequate role recognition | *“There is no philosophy of patting people on the back, saying thank you, well done, because you don't always need big money to satisfy people.” (P3)*  *“So I also identify with that, no. Probably there is also a, let's say, social dimension of role recognition that has gone missing.” (P6)*  *“While on the medical side there is a perceived difference between the figure of the medical executive who makes certain decisions at a high level, on our side that is missing, that is, the influence we can have on provided care.” (P18)* |
| ***Exo-level*** | Lacks in tailored education and professional development opportunities | *“So when interest is lacking, then you don't even find a way and manner to do it basically.” (P11)*  *“Courses offered at the local level... do not always respond to my interest.” (P11)*  *“Courses for coordinators have not been organized.” (P19)*  *“You have people who have to do the mandatory training and they don't come...and they fall behind because they don't get sent to the courses because there's always a bigger issue. Here, the perception is that there is no ability to properly schedule work within wards and, therefore, you live in the contingency of the day-to-day.” (P19)*  *“A great variability of tools, as I mentioned, maybe not be adequate to be able to take note of everything.” (P20)*  *“Few training tools.” (P20)*  *“They fail to keep up to date by reading articles of scientific literature.” (P21)* |
|  | Lack of independence in decision-making | *“What I regret is that we as coordinators didn't have a say for example on deciding what we should do and what we should not do.” (P2)*  *“Why can’t I have a say in this?” (P2)*  *“In short, when working, you shouldn't be done something that has not been shared... either way it doesn't set in motion that virtuous relationship between a valued and appreciated employee and one who is just called to ask him to do something.” (P2)*  *“Still don't completely have the autonomy to decide... because they still want to interfere.” (P18)*  *“But here it's difficult precisely because the departments are very large and then because the role is not yet structured in such a way that these department coordinators can be made operational, because they, in order to be able to perform this kind of role, should have the dashboards of the whole department in their hands.” (P17)* |
| ***Meso-level*** | Lack of structural and organizational support | *“Then I always go tripping over the fact that we need to have the right tools in order to deliver quality care. If we don't have the tools, you can't provide quality.” (P2)*  *“...leadership role that is actually provided and delivered... supportive toward employees...” (P16)*  *“A coordinator... needs to be supported.” (P17)*  *“...meeting once a month... instead... more support...” (P17)*  *“Actually we also have very little time and we do not guarantee that type of sharing and support to the coordinators in the operational units.” (P17)*  *“...it always seems like somebody that is very far away.” (P18)*  *“This culture of organization is kind of lacking, there are also some lacks, in my perception, in the continuity between what is happening in one's own care area, let's say, and what is happening in the other contexts, that is, there is perhaps a failure to grasp the needs that others have as well.” (P19)*  *“Then another big problem is that in my opinion we don't know how many things we omit because, I tell you, we really don't have tools that make our performance measurable. Then again, we are, as I told you, in a very self-referential environment.” (P20)*  *“We really lack tools to record our activity.” (P20)*  *“The other problem is that I think there is a lack of supports, organizational supports.” (P20)*  *“The coordinators don't have a real introduction process... the shortcomings, the omissions can be variable depending on how much support they have been receiving each day.” (P20)* |
|  | Lack of coordination | *“One can't tell [the patient], 'Look I talked to your sister yesterday'... in my opinion [this] can give a bad feeling about the final outcome.” (P2)*  *“There is no interaction between any of us. Each coordinator does it his own way. You don't know what happens with one or the other.” (P3)*  *“Well, the lack of communication is given, I tell you, by the vortex, by the phone, but not everything can be aimed at me, but them.” (P12)*  *“There are people who communicate and people who do not communicate.” (P19)*  *“Meetings… are organized, not as frequently as I would consider appropriate… many issues, in my opinion, should be handled as soon as possible.” (P22)* |
| ***Micro-level*** | Ambiguities in the role expectations | *“Then if you want you can ask me what activities are not consistent with my coordinating role and I do anyway.” (P7)*  *“Because my coordinating role is combined with my role as a nurse educator. So let's say that when performing activities within this organization, I'm not just a coordinator or a nurse educator, I'm both.” (P7)*  *“Not being present in the classroom and… 'shaping' their nursing thinking, was a bit of an issue.” (P8)*  *“There is a poor perception on their behalf of the role they have in a context that has changed a lot from the way it was 20 years ago.” (P19)*  *“It’s as if the coordinator is perceived more as the manager… acting just as a liaison, being an intermediate figure, perhaps one would expect… that he would also be closer to those on the front line.” (P22)* |
|  | Bureaucratic burden | *“I can't lose such an important person, a person with a qualification, with a responsibility to do inventories.” (P2)*  *“To order a medication that I need in 6 hours, in 24 hours, in 48 hours, I have to waste, I won't say two hours, but a ton of time. Because of this. This seems almost scientific to me. We analysed the reason why. Because the procedures were extremely slow, they were complicated, the system did not support the whole procedure. This means you don't give me appropriate tools.” (P2)*  *“A gradual but massive increase now of burocratic activities that... have been taken away from other structures... they are under the coordinators.” (P4)*  *“As for the bureaucratic-administrative dimension, I'll tell you that actually what is missed is due to the fact that this dimension is just a lot, it's too much and it's often not consistent with the goals it's designed for.” (P13)*  *“Perhaps because we are so overburdened with bureaucratic activities, we have to answer to our management, we have to update certain things.” (P17)* |
|  | Lack of team cohesion and loneliness | *“Perhaps there is not even a willingness to share and face... some issues that are related precisely to the actual loneliness of the managerial role.” (P6)*  *“You can have super professionals and still have a department that is not working... because the team is missing.” (P8)*  *“Coordinators… don’t have the opportunity to have an inclusion project.” (P17)* |
|  | Disjointed information flows | *“The fact that we know more and more have different access points to take information... this thing here is not very clear and so there is not a clear overall project.” (P20)*  *“We have little benchmarking with others, with other coordinators within the department, with other coordinators within the company, so we also learn little of others.” (P20)* |
| ***Nurse manager level*** | Chronic time scarcity and priority skills | *“Because I don't have the time. 11 hours a day and then I'm exhausted.” (P1)*  *“You cannot give an individual, in this case a coordinator, a workload that is mathematically impossible to bear.” (P2)*  *“I think that many coordinators no longer have the ability to manage their own time because they are oppressed by so many things.” (P2)*  *“No one ever has time. It can't be done.” (P2)*  *“No these sophisms are not sophistry, because in my opinion I have always thought that a group must have a good working environment to be able to work well. Elementary, Watson, you would say. And how do you get that when one already arrives in the morning with a bag full of backlog?” (P2)*  *“I already know that tomorrow morning I won't be able to do them [evaluations]. So, I can even feel this burden from home.” (P2)*  *“You neglect a little bit of this… training… before you send someone for training, you need to guarantee shift coverage.” (P3)*  *“Then it is true that there are also things, i.e., a super pressed academic calendar, even though by now we all know that in four years they would all be handled better, the same things would probably be handled with even more relaxed timelines for everyone.” (P9)*  *“Let me give you an example. I had asked second-year's students to prepare something for the mid-term evaluation, that is, to think about a patient to take to me for the mid-term evaluation, with the idea that we could discuss it for half an hour. However, it is clear that doing this means devoting time to the mid-term evaluation, at least half an hour, forty minutes for each student. And then there is the chat with the clinical tutor, which in some cases I know is useful, in others less so, however everyone expects to have a discussion with you. In addition to all this then you have the technical time of moving from one location to another, in my case, and so on. So it becomes an activity that you eventually give up.” (P10)*  *“Just the revision of the curriculum, the syllabus or the lectures... I can spend less time on.” (P11)*  *“I follow 3, 2, 4 students a year... otherwise I wouldn’t be able to.” (P11)*  *“Yes, as for this I could tell you it's because of a lack of time.” (P13)*  *“You have to distinguish between those that I prefer to postpone and those that I actually postpone.” (P15)*  *“Meetings with patients, with family members, clinical management of symptoms, you can't postpone them, and anyway you have 7-8 hours in the day, you can't manage more than that, in short.” (P15)*  *“It is clear that there are some situations in which even postponing can be useful to let the situation develop, in other cases it would be more useful to do it right away, and instead, due to contingent activities during the day, I am not always able to follow this strategy.” (P15)*  *“On the other hand, one cause could be, but I guess, that the coordinator is too overburdened with some activities, maybe mismanaged? in his working time and that, therefore, actually take away useful time for him to also be able to devote himself to a whole other set of activities that are closer to us, but bureaucratic in nature I think. One example, shift management which takes away so much time, which requires constant reshuffling, yes, but again I think it starts a little bit from a nursing coordinator's own organization on how they want to manage their shifts, how they want to manage the desires of their staff, etc., etc.” (P16)*  *“They omit or delay activities because they lack time. So whenever there's an increase in demands, they have to establish priorities.” (P20)*  *“Activities are concentrated in a very narrow time frame, hence the lack of time, as I was telling you.” (P20)*  *“I don't have time to do this.” (P21)*  *“Middle management roles, in my opinion, are so overwhelmed by the day-to-day, that they can't do planning, they can't have clean, critical thinking with respect to a little bit higher goal. Let's say you have to spend a lot of time getting people to understand the goodness of taking some paths that may seem more theoretical, because it seems like the day-to-day is looming and, therefore, sometimes they tell you.” (P21)* |
|  | Lack of experience | *“I'll give you an example, which is something I have experienced recently, the very fact of having many many many young colleagues who are inexperienced brings tension within the group, and this is felt at all levels, you know.” (P4)*  *“In addition, there's also the fact that this group is changing fast and, therefore, support for new hires is also needed, they have to be flanked, also with respect to the emotional dimension.” (P15)*  *“In my opinion, the reason, when I think of the department director, SOC (Complex Operational Structure) director, I think of a professional who is very experienced in clinical care and, therefore, in addition to developing the organizational, management dimension, they are usually also university professors, who then have all the training and research activities and then have a lot of clinical experience. If I think of directors, they are usually the most experienced clinicians within a team. Instead from us, and it's not criticism, I mean it's a consideration, instead from us, for example, if I think of my department head [the interviewee uses an acronym], but also my coordinator, of course a call is issued and in my opinion it is not recognized the important, that is, maybe the importance that even the manager and the coordinator have an impact on the clinic, this is not understood. That for God's sake, I mean she is very good, when we are in trouble she helps us take blood samples from (***typology of patients**), she comes to us, she answers the bells, so really I can't say anything against her, meaning that...however she lacks clinical experience at our level, I mean as a nurse, as a professional, let's say. Same thing for managers, meaning that, in my opinion, it is very difficult to have influence in a clinical setting, if you don't have clinical experience.” (P18)*  *“This means an absolute inability...poor perception, I would even say inability, to ensure the role, which is instead to govern resources and to understand that something like this then causes you cascading failures...it's hard. Let's say that the perception that we have, that (***name of service**) has is that people have kind of improvised to perform this role of coordinators and they don't always have the perception of what are the consequences of their missed actions.” (P19)*  *“Some people don't have those features; some people maybe just can't do it because they don't have the skills.” (P19)*  *“Now it is different since I have managers who are 29... Very good... but lacking experience.” (P21)* |
|  | Attitudes | *“Meaning that everyone tends to only care about their business without actually caring about what's going on elsewhere.” (P19)* |
|  | Sense of disengagement | *“Perhaps a slight disaffection in a system that is putting a strain on you.” (P3)*  *“Here, if I have to say one of the issues that you can find, then I'm going speak off the cuff, excuse me, one of the issues that you can find at the association level, but maybe the same thing that you find at the corporate level, hospital level, that is working, there is a sort of disaffection of people, little interest, to engage.” (P3)*  *“Perhaps it is not only a being burdened by doing so many things, it can also be a choice not to do certain things because one doesn't feel like doing it, with respect to those things for which one can say “no I don't want to do this one, this one bothers me, whatever, let someone else do it or why do I have to do it.” It can also be an arbitrary choice to decide to omit something.” (P17)* |

**Legend:** P, Participant.
